# Supplementary material for: The genome sequence of Vicia sativa L., 1753 (Fabales: Fabaceae)
Source: Wellcome Open Res. 2026 Jun 3;11:339. [Version 1] doi: 10.12688/wellcomeopenres.26708.1 (PMC13550887; doi:10.12688/wellcomeopenres.26708.1)
Supplement: Peer review history

# Reviewer report: Reviewer response for version 1

**DOI:** 10.21956/wellcomeopenres.29409.r158159

**Published:** 2026-07-06

**Authors:** Fabrício Almeida-Silva

**Affiliations:** 1 VIB Center for Plant Systems Biology, Ghent, Belgium

In this manuscript, Christenhusz et al. describe the genome of the legume Vicia sativa, produced as part of the Darwin Tree of Life project. Data were produced, processed, and analyzed using state-of-the-art software tools typically used in genome projects of this scale (such as ERGA, Earth BioGenome Project, etc). As a consequence, the obtained genome assembly and annotation are of very high quality. The genome will be very valuable to the community, especially to those studying genome evolution in legumes. Therefore, I believe this manuscript can be accepted in its current form.

Are sufficient details of methods and materials provided to allow replication by others?

Yes

Is the rationale for creating the dataset(s) clearly described?

Yes

Are the datasets clearly presented in a useable and accessible format?

Yes

Are the protocols appropriate and is the work technically sound?

Yes

Reviewer Expertise:

Evolutionary genomics, Systems Biology, Bioinformatics

I confirm that I have read this submission and believe that I have an appropriate level of expertise to confirm that it is of an acceptable scientific standard.

# Reviewer report: Reviewer response for version 1

**DOI:** 10.21956/wellcomeopenres.29409.r157514

**Published:** 2026-06-19

**Authors:** Nguyen Nhat Nam

**Affiliations:** 1 Tra Vinh University, Vinh Long, Vietnam

The current Data Note reports the full genome assembly of Vicia sativa L. as a part of the Darwin Tree of Life Project. This is a well-prepared manuscript with high-quality and valuable genome resources that meet current standards for genome notes. Therefore, in my opinion, it can be considered for acceptance for indexation. Only a minor correction should be addressed according to the inconsistent common name of Vicia sativa in the abstract (Narrowleaf Vetch) and introduction (Common Vetch).

Are sufficient details of methods and materials provided to allow replication by others?

Yes

Is the rationale for creating the dataset(s) clearly described?

Yes

Are the datasets clearly presented in a useable and accessible format?

Yes

Are the protocols appropriate and is the work technically sound?

Yes

Reviewer Expertise:

Plant genomics, comparative genomics, bioinformatics, and molecular biology

I confirm that I have read this submission and believe that I have an appropriate level of expertise to confirm that it is of an acceptable scientific standard.
